# Supplementary material for: HIV-1 Conserved Mosaics Delivered by Regimens with Integration-Deficient DC-Targeting Lentiviral Vector Induce Robust T Cells
Source: Mol Ther. 2017 Feb 22;25(2):494–503. doi: 10.1016/j.ymthe.2016.12.004 (PMC5368423; doi:10.1016/j.ymthe.2016.12.004)
Supplement: Document S1. Figure S1 and Table S1 [file mmc1.pdf]

YMTHE, Volume *25*

## Supplemental Information

# HIV-1 Conserved Mosaics Delivered by Regimens with Integration-Deficient DC-Targeting Lentiviral Vector Induce Robust T Cells

**Edmund G. Wee, Beatrice Ondondo, Peter Berglund, Jacob Archer, Andrew J. McMichael, David Baltimore, Jan H. ter Meulen, and Tomáš Hanke**

**Supplementary Table S1.** Immunization regimens

| | Grp | Regimen | n | Prime (Week 0) | | Boost (Week 3) | | Boost (Week 6) | |
|---|---|---|---|---|---|---|---|---|---|
| **Figure 2** BALB/c | 1 | Z | 4 | ZVex.tHIVconsv1 | 5x$10^{8}$ gc | - | - | - | - |
| | 2 | Z | 4 | ZVex.tHIVconsv1 | 5x$10^{9}$ gc | - | - | - | - |
| | 3 | Z | 4 | ZVex.tHIVconsv1 | 1x$10^{10}$ gc | - | - | - | - |
| | 4 | Z | 4 | ZVex.tHIVconsv2 | 5x$10^{8}$ gc | - | - | - | - |
| | 5 | Z | 4 | ZVex.tHIVconsv2 | 5x$10^{9}$ gc | - | - | - | - |
| | 6 | Z | 4 | ZVex.tHIVconsv2 | 1x$10^{10}$ gc | - | - | - | - |
| | | | | | | | | | |
| **Figure 3** BALB/c | 1 | ZZ | 5 | ZVex.tHIVconsv1 | 2.5x$10^{9}$ gc | ZVex.tHIVconsv1 | 2.5x$10^{9}$ gc | - | - |
| | | | | ZVex.tHIVconsv2 | 2.5x$10^{9}$ gc | ZVex.tHIVconsv2 | 2.5x$10^{9}$ gc | | |
| | 2 | ZM | 5 | ZVex.tHIVconsv1 | 2.5x$10^{9}$ gc | MVA.tHIVconsv3 | 5x$10^{5}$ PFU | - | - |
| | | | | ZVex.tHIVconsv2 | 2.5x$10^{9}$ gc | MVA.tHIVconsv4 | 5x$10^{5}$ PFU | | |
| | 3 | ZC | 5 | ZVex.tHIVconsv1 | 2.5x$10^{9}$ gc | ChAdOx1.tHIVconsv5 | 1.4x$10^{6}$ IU | - | - |
| | | | | ZVex.tHIVconsv2 | 2.5x$10^{9}$ gc | ChAdOx1.tHIVconsv6 | 1.4x$10^{6}$ IU | | |
| | | | | | | | | | |
| **Figure 4 a, b & d** BALB/c | 1 | ZZ | 5 | ZVex.tHIVconsv1 | 2.5x$10^{9}$ gc | ZVex.tHIVconsv1 | 2.5x$10^{9}$ gc | - | - |
| | | | | ZVex.tHIVconsv2 | 2.5x$10^{9}$ gc | ZVex.tHIVconsv2 | 2.5x$10^{9}$ gc | | |
| | 2 | ZM | 5 | ZVex.tHIVconsv1 | 2.5x$10^{9}$ gc | MVA.tHIVconsv3 | 5x$10^{5}$ PFU | - | - |
| | | | | ZVex.tHIVconsv2 | 2.5x$10^{9}$ gc | MVA.tHIVconsv4 | 5x$10^{5}$ PFU | | |
| | 3 | ZC | 5 | ZVex.tHIVconsv1 | 2.5x$10^{9}$ gc | ChAdOx1.tHIVconsv5 | 1.4x$10^{6}$ IU | - | - |
| | | | | ZVex.tHIVconsv2 | 2.5x$10^{9}$ gc | ChAdOx1.tHIVconsv6 | 1.4x$10^{6}$ IU | | |
| | 4 | CZ | 5 | ChAdOx1.tHIVconsv5 | 1.4x$10^{6}$ IU | ZVex.tHIVconsv1 | 2.5x$10^{9}$ gc | - | - |
| | | | | ChAdOx1.tHIVconsv6 | 1.4x$10^{6}$ IU | ZVex.tHIVconsv2 | 2.5x$10^{9}$ gc | | |
| | 5 | MZ | 5 | MVA.tHIVconsv3 | 5x$10^{5}$ PFU | ZVex.tHIVconsv1 | 2.5x$10^{9}$ gc | - | - |
| | | | | MVA.tHIVconsv4 | 5x$10^{5}$ PFU | ZVex.tHIVconsv2 | 2.5x$10^{9}$ gc | | |
| | 6 | CM | 5 | ChAdOx1.tHIVconsv5 | 1.4x$10^{6}$ IU | MVA.tHIVconsv3 | 5x$10^{5}$ PFU | - | - |
| | | | | ChAdOx1.tHIVconsv6 | 1.4x$10^{6}$ IU | MVA.tHIVconsv4 | 5x$10^{5}$ PFU | | |
| | | | | | | | | | |
| **Figure 4c** BALB/c | 1 | ZZ | 4 | ZVex.tHIVconsv1 | 2.5x$10^{9}$ gc | ZVex.tHIVconsv1 | 2.5x$10^{9}$ gc | - | - |
| | | | | ZVex.tHIVconsv2 | 2.5x$10^{9}$ gc | ZVex.tHIVconsv2 | 2.5x$10^{9}$ gc | | |
| | 2 | ZM | 4 | ZVex.tHIVconsv1 | 2.5x$10^{9}$ gc | MVA.tHIVconsv3 | 5x$10^{5}$ PFU | - | - |
| | | | | ZVex.tHIVconsv2 | 2.5x$10^{9}$ gc | MVA.tHIVconsv4 | 5x$10^{5}$ PFU | | |
| | 3 | ZC | 4 | ZVex.tHIVconsv1 | 2.5x$10^{9}$ gc | ChAdOx1.tHIVconsv5 | 1.4x$10^{6}$ IU | - | - |
| | | | | ZVex.tHIVconsv2 | 2.5x$10^{9}$ gc | ChAdOx1.tHIVconsv6 | 1.4x$10^{6}$ IU | | |
| | 4 | -M | 4 | - | - | MVA.tHIVconsv3 | 5x$10^{5}$ PFU | - | - |
| | | | | | | MVA.tHIVconsv4 | 5x$10^{5}$ PFU | | |
| | 5 | -C | 4 | - | - | ChAdOx1.tHIVconsv5 | 1.4x$10^{6}$ IU | - | - |
| | | | | | | ChAdOx1.tHIVconsv6 | 1.4x$10^{6}$ IU | | |
| | 6 | ZeZe | 4 | ZVex empty | 2.5x$10^{9}$ gc | ZVex empty | 2.5x$10^{9}$ gc | - | - |
| | | | | ZVex empty | 2.5x$10^{9}$ gc | ZVex empty | 2.5x$10^{9}$ gc | | |
| | | | | | | | | | |
| **Figure 5** CD1-Swiss | 1 | ZM | 7 | ZVex.tHIVconsv1 | 2.5x$10^{9}$ gc | MVA.tHIVconsv3 | 5x$10^{5}$ PFU | - | - |
| | | | | ZVex.tHIVconsv2 | 2.5x$10^{9}$ gc | MVA.tHIVconsv4 | 5x$10^{5}$ PFU | | |
| | 2 | CM | 7 | ChAdOx1.tHIVconsv5 | 1.4x$10^{6}$ IU | MVA.tHIVconsv3 | 5x$10^{5}$ PFU | - | - |
| | | | | ChAdOx1.tHIVconsv6 | 1.4x$10^{6}$ IU | MVA.tHIVconsv4 | 5x$10^{5}$ PFU | | |
| | 3 | ZCM | 7 | ZVex.tHIVconsv1 | 2.5x$10^{9}$ gc | ChAdOx1.tHIVconsv5 | 1.4x$10^{6}$ IU | MVA.tHIVconsv3 | 5x$10^{5}$ PFU |
| | | | | ZVex.tHIVconsv2 | 2.5x$10^{9}$ gc | ChAdOx1.tHIVconsv6 | 1.4x$10^{6}$ IU | MVA.tHIVconsv4 | 5x$10^{5}$ PFU |

gc – genome copies; IU – infection unit; PFU – plaque-forming unit

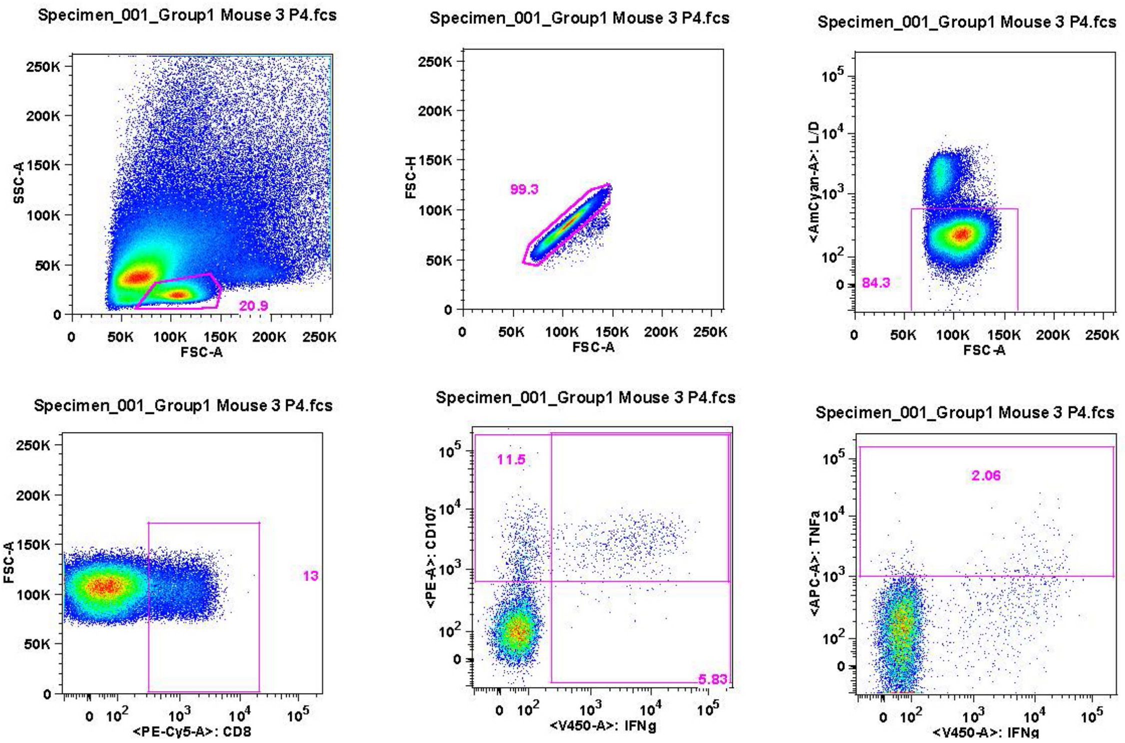


Supplementary Figure S1 Gating strategy for **Figure 3** Representative FACS plots show the gating strategy for assessing co-expression of IFN-γ, TNF-α, IL-2 and CD107a.
