# Supplementary material for: HIV-1 Conserved Mosaics Delivered by Regimens with Integration-Deficient DC-Targeting Lentiviral Vector Induce Robust T Cells
Source: Mol Ther. 2017 Feb 22;25(2):494–503. doi: 10.1016/j.ymthe.2016.12.004 (PMC5368423; doi:10.1016/j.ymthe.2016.12.004)
Supplement: Document S2. Article plus Supplemental Information [file mmc2.pdf]

Molecular Therapy
Original Article

HIV-1 Conserved Mosaics Delivered by Regimens with Integration-Deficient DC-Targeting Lentiviral Vector Induce Robust T Cells

Edmund G. Wee,[1,6] Beatrice Ondondo,[1,6] Peter Berglund,[2] Jacob Archer,[2] Andrew J. McMichael,[3] David Baltimore,[4] Jan H. ter Meulen,[2] and Tomáš Hanke[1,5]

[1]The Jenner Institute, University of Oxford, Oxford OX3 7DQ, UK; [2]Immune Design Corp., Seattle, WA 98102, USA; [3]Nuffield Department of Clinical Medicine, University of Oxford, Oxford OX3 7FZ, UK; [4]Division of Biology and Biological Engineering, California Institute of Technology, Pasadena, CA 91125, USA; [5]International Research Center for Medical Sciences, Kumamoto University, Kumamoto 860-8555, Japan

To be effective against HIV type 1 (HIV-1), vaccine-induced T cells must selectively target epitopes, which are functionally conserved (present in the majority of currently circulating and reactivated HIV-1 strains) and, at the same time, beneficial (responses to which are associated with better clinical status and control of HIV-1 replication), and rapidly reach protective frequencies upon exposure to the virus. Heterologous prime-boost regimens using virally vectored vaccines are currently the most promising vaccine strategies; nevertheless, induction of robust long-term memory remains challenging. To this end, lentiviral vectors induce high frequencies of memory cells due to their low-inflammatory nature, while typically inducing only low anti-vector immune responses. Here, we describe construction of novel candidate vaccines ZVex.tHIVconsv1 and ZVex.tHIVconsv2, which are based on an integration-deficient lentiviral vector platform with preferential transduction of human dendritic cells and express a bivalent mosaic of conserved-region T cell immunogens with a high global HIV-1 match. Each of the two mosaic vaccines was individually immunogenic. When administered together in heterologous prime-boost regimens with chimpanzee adenovirus and/or poxvirus modified vaccinia virus Ankara (MVA) vaccines to BALB/c and outbred CD1-Swiss mice, they induced a median frequency of over 6,000 T cells/10^6 splenocytes, which were plurifunctional, broadly specific, and cross-reactive. These results support further development of this vaccine concept.

INTRODUCTION

Vaccine protection against infection/disease requires targeting the causative microorganisms at their most vulnerable sites by robust and timely immune responses.[1,2] Robustness broadly sums the overall magnitude of responses and effectiveness of their protective functions, which have to be exerted at the right anatomical localization at the right time.[3,4] To blunt immune attacks, highly variable microorganisms evolved multiple evasive strategies, of which perhaps the most common employs so-called decoy epitopes.[5,6] These are easily accessible, highly immunogenic determinants that do not stop the microbes when targeted. This is because the most exposed decoy sites are in protein regions non-essential for survival, structure, or function and are, therefore, easily mutated to render the mounted responses ineffective.[7–9] In contrast, structurally and functionally important regions are frequently subdominant.[7–13] This is equally true for antibody and cytotoxic T lymphocyte (CTL) responses.

Natural infection by HIV type 1 (HIV-1) induces strong T cell responses; however, they fail to protect against progression to AIDS due to continued immune escape.[8,14–19] Thus, effective vaccines should direct T cells to the functionally and structurally conserved regions of HIV-1 proteins.[20–24] Although these regions often contain subdominant epitopes, these are less prone to immune escape due to a resulting loss of replicative fitness.[25–29] Such regions are common to most HIV-1 variants including transmitted/founder viruses offering the potential for deployment against diverse worldwide circulating strains as well as already escaped viruses reactivated from the latent reservoirs.[27] We have pioneered virally vectored T cell vaccines designed as conserved alternating-clade consensus sequences (HIVconsv, first generation)[23] and taken them into clinical evaluation as heterologous prime-boost regimens.[30–33] More recently, we enhanced this conserved-region approach by replacing the consensus sequences with a bivalent mosaic design (tHIVconsvX, second generation),[34] which computationally increases as much as possible the match of candidate vaccines to the currently worldwide circulating HIV-1 variants for maximum effector efficacy.[34–38] Furthermore, we adjusted the boundaries of conserved regions to cover the majority of conserved and, at the same time, beneficial CTL epitopes, which have been associated in HIV-1-positive, treatment-naive cohorts on four continents with low viral load and high CD4$^+$ T cell count.[34,39,40] Finally, each of the six tHIVconsvX immunogens tHIVconsv1–tHIVconsv6 has the six conserved regions organized in a unique order

Received 22 June 2016; accepted 1 December 2016;
http://dx.doi.org/10.1016/j.ymthe.2016.12.004.

[6]These authors contributed equally to this work.

Correspondence: Tomáš Hanke, The Jenner Institute, University of Oxford, Old Road Campus Research Building, Roosevelt Drive, Oxford OX3 7DQ, UK.
E-mail: tomas.hanke@ndm.ox.ac.uk

 © 2017 The Authors.
This is an open access article under the CC BY license (http://creativecommons.org/licenses/by/4.0/).

to minimize induction of T cell responses to junctions, which are not present in the HIV-1 proteome and are, therefore, an avoidable distraction to the immune response.[34]

Initially, the tHIVconsvX conserved mosaic immunogens were administered by a combination of non-replicating simian (chimpanzee) adenovirus and non-replicating modified vaccinia virus Ankara (MVA) vectors with or without plasmid DNA priming. We have demonstrated high immunogenicity of these heterologous prime-boost regimens in both healthy and HIV-1-positive adults.[31,33] Both adenoviruses and poxviruses prime T cells in a high-inflammatory environment, which results in a brisk expansion of polyfunctional effector T cells, but may lead to suboptimal induction of boostable central memory T cell populations, which are important for long-term protection.[41] Furthermore, heterologous prime-boost regimens using a low-inflammatory prime, such as DNA and dendritic cell (DC)-based vaccination, followed by a high-inflammatory boost induced superior memory T cell responses.[41] Thus, lentiviral vectors are attractive vaccine modalities for induction of robust T cell responses against infectious disease and cancer.[42–44] We evaluated a novel third-generation lentiviral vector designated ZVex, which selectively targets DCs via interaction of its Sindbis-virus-derived envelope protein with the receptor DC-specific intercellular adhesion molecule (ICAM)-3 grabbing non-integrin DC-SIGN (CD209) expressed on immature DCs and is currently being advanced in cancer immunotherapy trials. ZVex is an HIV-1-derived, self-inactivating vector with the additional safety feature of integration deficiency, which is achieved by the combination of integrase inactivation and extended 3′ deletion of the vector backbone.[45,46]

In the present work, ZVex-vectored vaccines expressing a complementing pair of tHIVconsvX bivalent conserved mosaic immunogens designated ZVex.tHIVconsv1 and ZVex.tHIVconsv2 demonstrated high T cell immunogenicity alone and in heterologous prime-boost regimens with vaccines ChAdOx1.tHIVconsv5+ChAdOx1.tHIVconsv6 vectored by engineered chimpanzee adenovirus Y25 and MVA.tHIVconsv3+MVA.tHIVconsv4.[34] Taken together, these results support development of a heterologous prime-boost regimen using ZVex- and ChAdOx1- and/or MVA-vectored vaccines expressing conserved HIV-1 T cell epitopes for clinical application.

RESULTS

Design and Construction of the ZVex.tHIVconsv1 and ZVex.tHIVconsv2 Vaccines

Design of the six bivalent conserved mosaic tHIVconsvX immunogens of 872 amino acids (Figure 1A) was described previously.[34,47] For vaccinations, the mosaic pairs are administered together as tHIVconsv1+tHIVconsv2, tHIVconsv3+tHIVconsv4, and tHIVconsv5+tHIVconsv6. Note that in each immunogen, the individual regions are in different orders to avoid possible induction of strong, but irrelevant, responses to potential junctional epitopes.

The integration-deficient lentivector platform ZVex was described previously.[46] The open reading frames coding for conserved immunogens tHIVconsv1 and tHIVconsv2 (Figure 1A) were first individually inserted into the transfer vector genome and novel vaccines ZVex.tHIVconsv1 and ZVex.tHIVconsv2 were then prepared by a co-transfection of five distinct plasmid DNAs: the transgene-coding lentivector genome, the modified Gag/Pol packaging plasmid, two separate plasmids expressing the accessory proteins Rev of HIV-1 and Vpx of SIVmac, and a plasmid expressing a modified Sindbis virus glycoprotein.

The mosaic pairs of vaccines were delivered by heterologous regimens combining lentivirus vectors, simian adenovirus, and poxvirus MVA (Figure 1B). Sequential administrations of heterologous vectors avoid buildup of anti-vector immunity, which dampens induction of CTLs against the transgene products.[48–50]

ZVex.tHIVconsv1 and ZVex.tHIVconsv2 Vaccines Are Immunogenic in Mice

The initial confirmation of immunogenicity of each of the constructed vaccine components separately was carried out in mice. Elicited HIV-1-specific T cells were measured in an interferon (IFN)-γ enzyme-linked immunospot (ELISPOT) assay.[34] Mosaic proteins differ in about 10% of amino acids and the 401 unique 15-mer peptides overlapping by 11 amino acids (15/11) derived from both mosaics 1 and 2 were assembled into 10 peptide pools P1–P10, in which paired variant peptides were always in the same pool; this allowed us to sum the individual pool frequencies of responding cells to calculate total magnitudes of the response for each animal.[34]

Increasing doses of the individual ZVex.tHIVconsv1 and ZVex.tHIVconsv2 vaccines ranging from 5×10^8 to 1×10^{10} genome copies (gc) per dose (Table S1) were administered to the BALB/c mice. The gc-to-infectious unit ratio is consistently between 10:1 and 30:1. Our previous results in the H-2^d haplotype indicated that peptide pools P1 and P4 contained the most dominant epitopes detecting approximately 87% of the total response;[34] therefore, for simplicity, only the P1 and P4 pools were used in most immunologic readouts. Thus, single administrations of ZVex.tHIVconsv1 and ZVex.tHIVconsv2 induced specific T cell frequencies with medians of 570 and 193 spot-forming units (SFU)/10^6 splenocytes for P1 and exceeding 1,200 and 163 SFU/10^6 splenocytes for P4, respectively, at the intermediate dose of 5×10^9 gc (Figure 2). This dose was chosen as the total dose for further experiments.

T Cell Induction by ZVex Vaccines Is Augmented by Heterologous Boost

Induction of T cells against the conserved HIV-1 regions was assessed in homologous ZVex-ZVex and heterologous ZVex-MVA and ZVex-ChAdOx1 regimens (note that each ZVex, MVA, and ChAdOx1 administration delivers two mosaic vaccines each; see Figure 1B and Table S1). While the IFN-γ ELISPOT assay indicated high T cell frequencies induced by the homologous ZVex-ZVex regimen with medians of 775 and 1,675 SFU/10^6 splenocytes detected by peptide pools P1 and P4, respectively, the ZVex

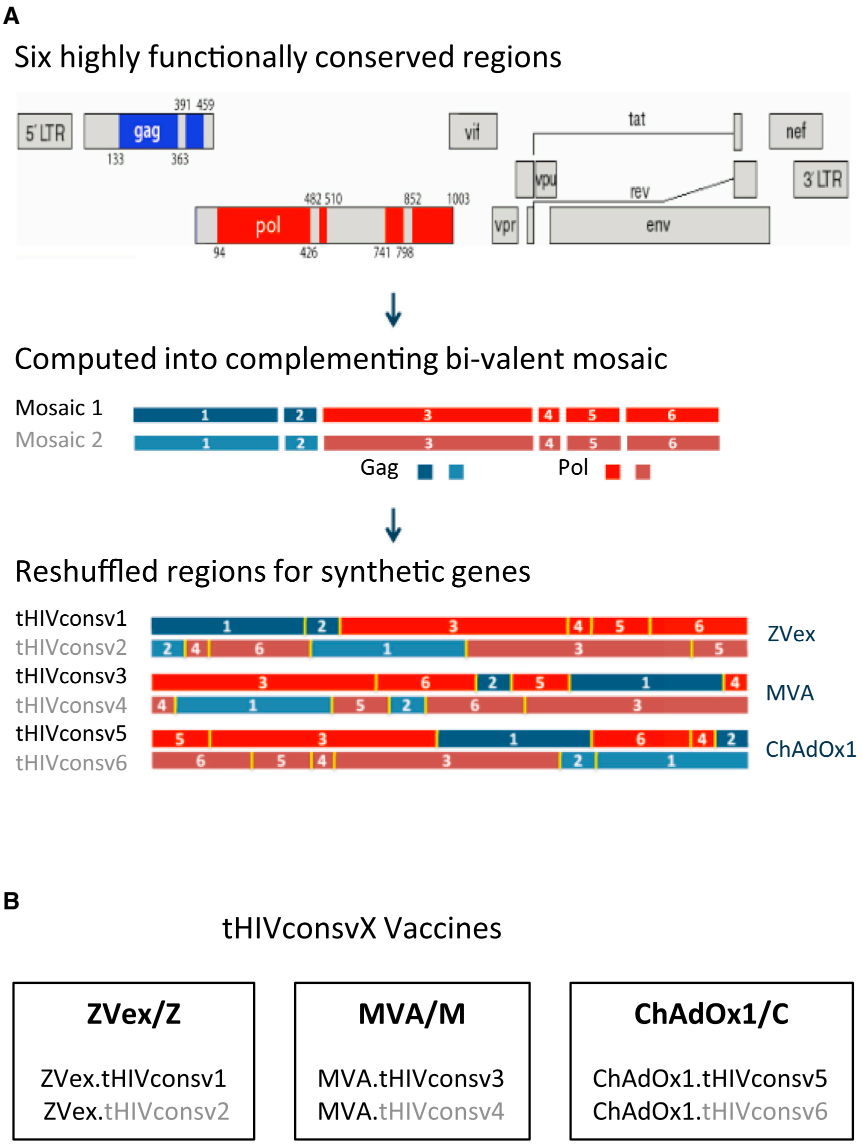

Figure 1. Vaccines

(A) tHIVconsvX immunogens (insets). The vaccines focus T cells on six highly functionally conserved regions of HIV-1 Gag and Pol proteins and their efficacy is enhanced by maximizing the vaccine match to global HIV-1 isolates through a computer-designed bivalent mosaic[47] and inclusion of beneficial epitopes.[39,40] For each immunogen tHIVconsv1-tHIVconsv6, the selected conserved regions are organized in different orders to avoid boosting of potential junctional epitopes. Mosaic 1 and mosaic 2 are always used together for each administrated dosing. (B) The six vaccine components used in this work: tHIVconsv1 and tHIVconsv2 are delivered by integration-deficient lentivirus vector ZVex, tHIVconsv3 and tHIVconsv4 are delivered by non-replicating poxvirus MVA, and tHIVconsv5 and tHIVconsv6 are delivered by non-replicating simian (chimpanzee) adenovirus ChAdOx1.

combination with heterologous MVA and ChAdOx1 boosts for both regimens reached similar median frequencies of 1,525 and 5,500 SFU/10^6 splenocytes for the two respective P1 and P4 pools (Figure 3A). Thus, priming with ZVex and boosting with poxvirus MVA or simian adenovirus ChAdOx1 strongly enhanced T cell responses to conserved regions.

Plurifunctionality of the vaccine-elicited $CD8^+$ T cells in terms of IFN-γ, tumor necrosis factor (TNF)-α, and interleukin (IL)-2 production and degranulation, the equivalent of killing measured by surface expression of CD107a, was assessed using a polychromatic flow cytometry. The relative inter-regimen percentages of specific T cells correlated well with the IFN-γ ELISPOT assay. For the strongest peptide pool P4, the T cell frequencies detected for the ZVex-MVA regimen reached medians of 13.4%, 12.1%, 1.2%, and 13.0% responding cells of the total $CD8^+$ T cells in the spleen for IFN-γ, TNF-α, IL-2, and CD107a, respectively (Figure 3B). Responses to pool P1 were less than one-third of those to P4. The heterologous prime-boost regimens again induced the highest plurifunctional responses (Figure 3C).

Conserved Mosaic-Induced T Cells Recognize Variant HIV-1 Peptides

Next, we tested comprehensively heterologous vaccine regimens and used the generated T cell responses to assess the "depth" of recognition of epitope variants induced by the bivalent mosaic immunogens. First, we tested five regimens involving lentivirus vectors ZVex-ZVex, ZVex-MVA, ZVex-ChAdOx1, ChAdOx1-ZVex, and MVA-ZVex and compared their immunogenicity with that of our currently clinically pursued ChAdOx1-MVA combination. Thus, using the immunodominant peptide pool P4 in the IFN-γ ELISPOT assay, the two strongest and statistically inseparable from each other were the ZVex-MVA and ChAdOx1-MVA regimens; frequencies detected by pool P1 were lower than those to P4 and similar among regimens (Figure 4A). The overall trend of the regimens' relative hierarchy for induction of IFN-γ was reproduced by the intracellular cytokine staining analysis (Figure 4B). Using all 10 pools P1–P10 covering 15/11 peptides across the six conserved regions of the two mosaic immunogens, we also demonstrated the role of the ZVex-vectored vaccines in priming responses for ChAdOx1 and MVA, which was most obvious for the immunodominant pool P4 (Figure 4C). This experiment also indicated that the homologous empty vector ZVex-ZVex regimen induced responses to conserved HIV-1 Gag pools P1 and to a lesser extent P2, with median 613 and 72 SFU/10^6 splenocytes, respectively (Figure 4C).

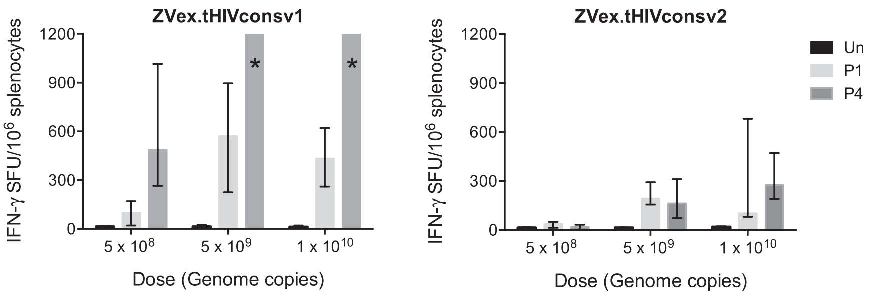

Figure 2. Dose Response for ZVex.tHIVconsv1 and ZVex.tHIVconsv2

Groups of BALB/c mice were immunized with increasing doses of either ZVex.tHIVconsv1 or ZVex.tHIVconsv2 vaccine alone administered intramuscularly (Table S1). Immune splenocytes were tested in an IFN-γ ELISPOT assay unstimulated (Un) or stimulated with peptide pools P1 or P4. Frequencies of conserved-region-specific T cells are shown as the median and interquartile range (n = 4).

To assess the potential for cross-recognition of diverse HIV-1 isolates following the bivalent mosaic immunization, we examined the tHIVconsv1+HIVconsv2-elicited major histocompatibility complex (MHC) class I-restricted responses to epitopes AMQ (AMQMLKETI; pool P1) and VLV (VLVGPTPVNI; pool P4), which are on the top of the $CD8^+$ T cell H-2^d-restricted hierarchy of the conserved mosaic regions.[34] For each epitope, we used the peptide pool, the two most stimulatory 15-mer peptides in the pool encompassing the minimal epitope, as well as four minimal peptide variants found in natural HIV-1 isolates present in the Los Alamos National Laboratory HIV Sequences Database (LANL-HSD). Overall, regimens did not influence the relative SFU among tested variant peptides and the overall pool SFU magnitudes were reflected by the relative magnitudes of individual peptide responses (Figure 4D). The minimal index peptides tended to yield higher frequencies compared to their parental 15-mer peptides, which require processing prior to MHC binding, and their other subdominant variants. Thus, for the weakest AMQ of the two epitopes, three of four tested minimum peptide variants were recognized and peptide variant AMELFLKDTA was not recognized at all. For the strongest VLV epitope of pool P4, four of four variants were recognized. Thus, the recognition of mutated epitopes induced by the bivalent mosaic extended beyond the two variants present in the tHIVconsv1 and tHIVconsv2 immunogens.

ZVex-Vectored Vaccines Induce Robust and Broadly Specific T Cells in Outbred Animals

Measurements of specific T cells have been aided by the use of isogenic mouse strains, which greatly simplify the immunologic readouts and decrease the inter-animal variation and hence animal numbers. However, an outbred mouse stock provides more rigorous and realistic tests for vaccine immunogenicity. For these reasons, we used outbred mouse stock CD1-Swiss for comparison of the ZVex-MVA, ChAdOx1-MVA, and ZVex-ChAdOx1-MVA regimens. In this experiment, we enumerated the vaccine-elicited T cells in an IFN-γ ELISPOT assay again using all 10 pools P1–P10. Overall, the vaccination induced broadly specific T cells with a median (range) of 6 (4–6), 3 (0–7), and 6 (2–8) of recognized peptide pools with T cell frequencies above 50 SFU/10^6 splenocytes for the ZVex-MVA, ChAdOx1-MVA, and ZVex-ChAdOx1-MVA regimens, respectively (Figure 5). The total magnitudes reached a median (range) of 6,280 (450–8,320), 3,730 (0–8,600), and 6,040 (170–16,120), respectively, which were statistically indistinguishable. These results suggest a good range of responsiveness in outbred animals and support further development of the ZVex.tHIVconsv1 and ZVex.tHIVconsv2 vaccines toward clinical use.

DISCUSSION

In the present work, we describe construction of two novel components of a candidate anti-HIV-1 T cell-based vaccine used together in a heterologous prime-boost regimen. The design combines conserved-region bivalent mosaic T cell immunogens with a uniquely high match to global HIV-1 isolates[34] and the novel integration-deficient lentivirus vector ZVex, which combines multiple safety features, including integration deficiency, with efficient targeting and transduction of human dendritic cells.[46] The two novel vaccines ZVex.tHIVconsv1 and ZVex.tHIVconsv2 induced robust, variant cross-reactive, and plurifunctional T cell responses, which were broadly specific in outbred mice. The potency of the ZVex-MVA regimen for $CD8^+$ T cell induction was as good as that of the currently pursued ChAdOx1-MVA. These attributes meet many desired features of candidate HIV-1 vaccines, which should progress to clinical evaluation.

The bivalent mosaic design of HIV-1 T cell vaccines represents the second generation of conserved immunogens[34] and follows the first generation of alternating-clade consensus sequences.[31] The second-generation immunogens combine the three currently most advanced approaches for T cell vaccines to tackle HIV-1 diversity and escape, whereby the efficacy of refocused $CD8^+$ T cells on the structurally and functionally constrained regions of HIV-1 proteins (mutations that result in fitness loss[25–28]) is further enhanced by the currently superior vaccine match to global HIV-1 variants of the bivalent mosaic[34,35,47] and inclusion of beneficial T cell epitopes.[34,39,40] Here, we demonstrated that these conserved mosaic immunogens can be efficiently presented to the murine immune system by the ZVex platform, which was highly immunogenic on its own and combined well with the ChAdOx1- and MVA-vectored vaccines currently lined up for clinical evaluation. Elicited T cells recognized several variants of the two investigated epitopes, including those not present in the immunogens.

The traditional vaccine approach of live attenuation is currently not considered safe for HIV-1, and subunit vaccines have to date not induced sufficient protection as stand-alone modalities, despite the use of novel adjuvants.[51–53] Thus, non-replicating viral vectors including derivatives of human and simian adenoviruses and

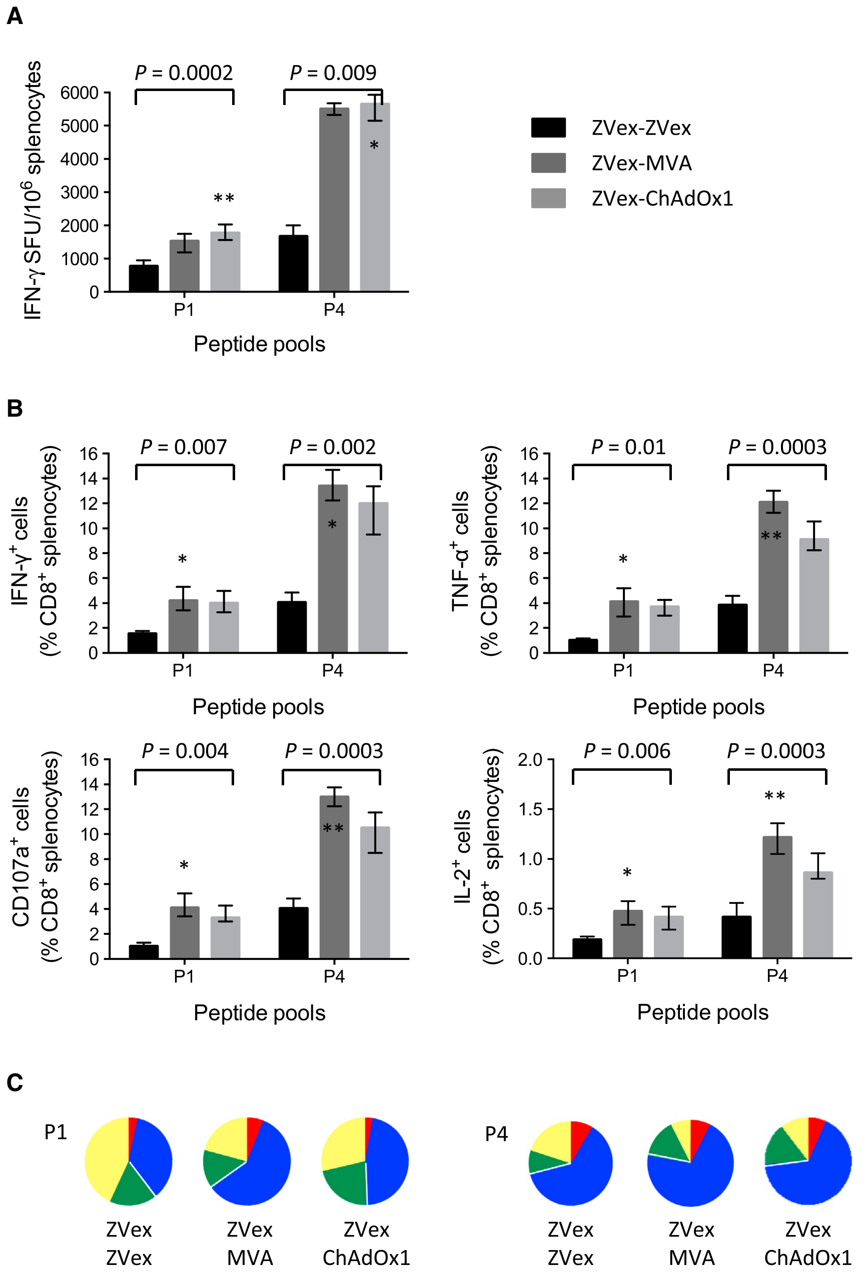

Figure 3. Functionality of HIV-1-Specific CD8$^+$ T Cells Induced by Prime-Boost Regimens

Groups of BALB/c mice were immunized using prime-boost regimens of ZVex, MVA, and ChAdOx1 (note that each vaccine modality delivered both mosaic 1 and mosaic 2 together; see Table S1) and were euthanized 9 days later. Vaccine-elicited T cells were enumerated in an (A) IFN-γ ELISPOT assay or (B) intracellular cytokine staining assay determining the fractions of cells producing intercellular signaling molecules IFN-γ, TNF-α, and IL-2 and degranulating (CD107a) upon restimulation with peptide pools P1 or P4. See Figure S1 for the gating strategy. Specific T cell frequencies are shown as the median and interquartile range (n = 4). The Kruskal-Wallis test (ANOVA) was used for each peptide pool to determine the approximate *P* values shown above the graph followed by multiple comparisons of vaccine groups Z-Z versus Z-M, Z-Z versus Z-C, and Z-M versus Z-C corrected by the Dunn test. As none of the differences between Z-M versus Z-C were significant, asterisks indicate significance in the first two comparisons (*p < 0.05; **p < 0.01). (C) The pie charts indicate the plurifunctionality of vaccine-elicited tHIVconsvX-specific T cells: yellow, one function; green, two functions; blue, three functions; and red, four functions.

poxviruses, as well as DNA, alphavirus replicons, adeno-associated viruses, and others are being studied because of their capacity to induce integrated immune responses consisting of antibody and T cells, which, when effective, are believed to be critical for prevention and control of HIV-1 infection.[21,22,54,55] Since repeated vaccination with homologous vector induces strong anti-vector immunity, which blunts responses against the transgene product, prime-boost regimens with heterologous vectors are generally viewed as superior.[21,56–58] In the past years, lentiviral vectors have increasingly been explored as vaccine platforms for cancer and infectious diseases because of their capability to transduce non-dividing antigen-presenting cells, directly prime CD8$^+$ T cells, and induce robust T cell memory responses due to their low-inflammatory mode of action and prolonged low-level antigen production.[43] Lentivirus-based vaccines expressing structural and non-structural HIV-1 proteins induced robust immune responses in preclinical animal models and partially protected non-human primates against simian immunodeficiency virus (SIV) challenge.[59] In addition, heterologous immunization with a lentiviral vaccine and human adenovirus vector HAdV-5 expressing HIV-1 Gag, Pol, and Rev proteins resulted in superior immunogenicity and overcame pre-existing anti-HAdV-5 immunity.[60] While the majority of these experiments were performed with integrating lentiviral vectors pseudotyped with the envelope glycoprotein of vesicular stomatitis virus VSV-G, giving these vectors a broad tropism, recent developments have explored integration deficiency as an important safety feature and targeting of antigen-presenting cells as a means to modulate immune responses.[61,62]

ZVex is a third-generation lentiviral vector targeted to professional antigen-presenting cells through the selective interaction of an engineered Sindbis virus envelope with DC-SIGN expressed on immature DCs.[63] To overcome the SAMHD-1-mediated resistance of human dendritic cells to lentivirus infection, the accessory protein SIVmac Vpx was incorporated in ZVex to improve transduction efficacy.[64]

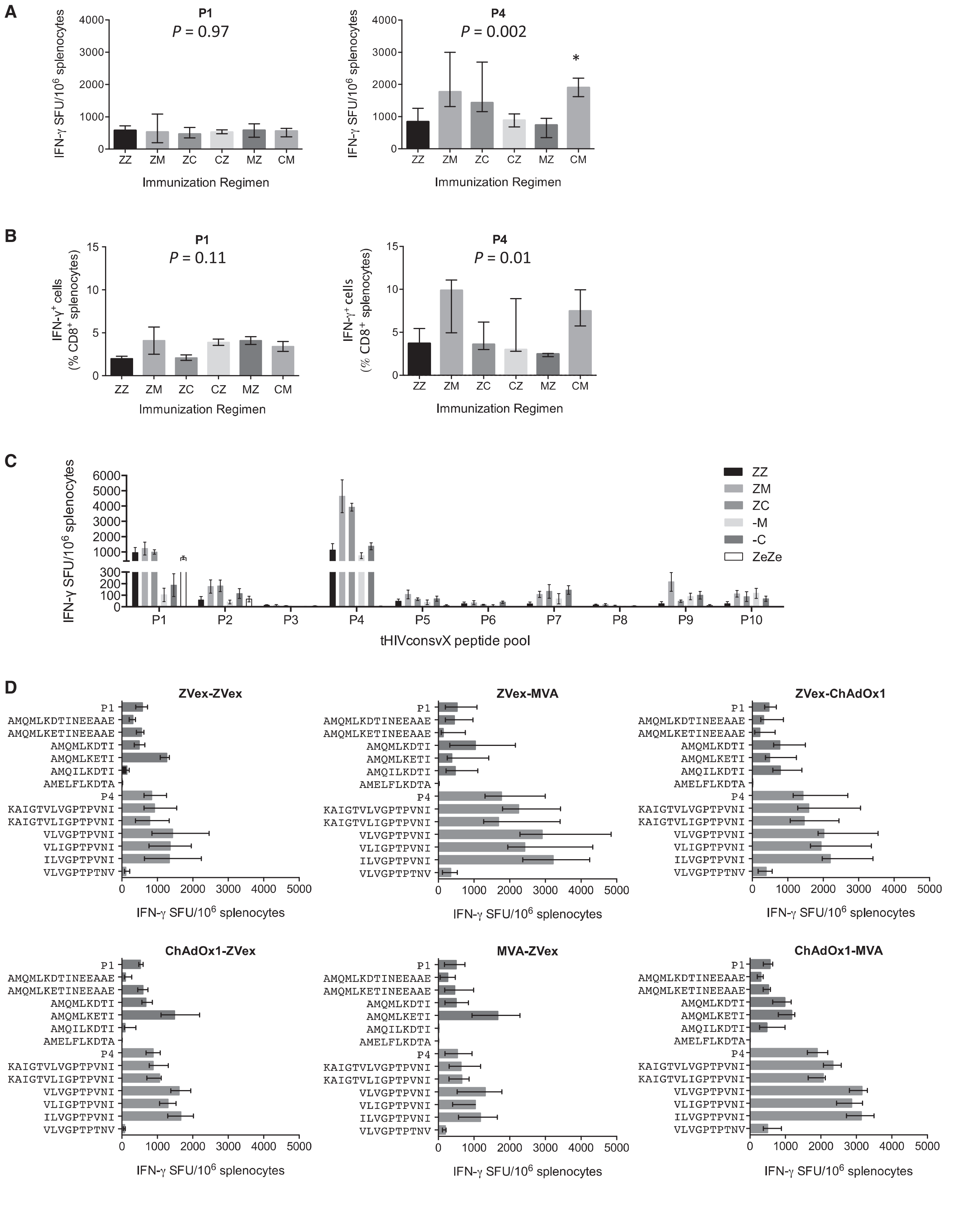

(legend on next page)

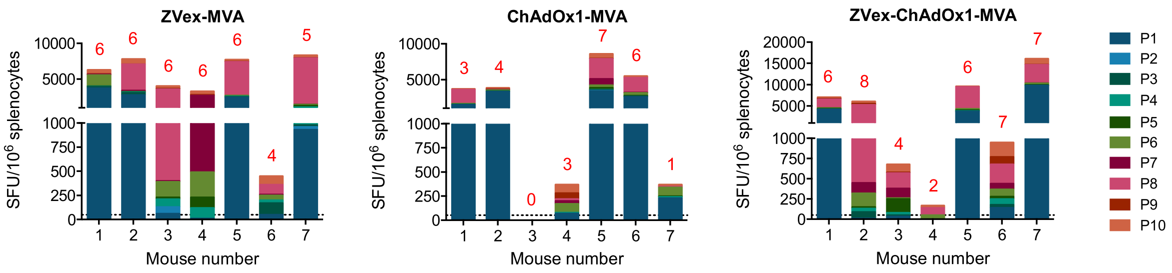

Figure 5. Broad Specificity of tHIVconsvX-Elicited T Cells for Conserved Epitopes

Groups of seven outbred CD1-Swiss mice were immunized using the ZVex-MVA, ChAdOx1-MVA, or ZVex-ChAdOx1-MVA regimens (note that each vaccine modality delivered both mosaic 1 and mosaic 2 together: see Table S1) and were euthanized 1 week later. Isolated splenocytes from individual mice were tested in an IFN-γ ELISPOT assay against 10 pools P1–P10 of 15/11 peptides across the full length of all six tHIVconsvX conserved regions. Average frequencies of triplicate wells are shown for each mouse and pool. Numbers above each animal composite column give the number of peptide pools recognized by vaccine-elicited T cells with frequencies above 50 SFU/10^6 splenocytes.

Integration deficiency of ZVex was achieved through genetic inactivation of the integrase (D64V mutation) and extended deletion of the 3′ region of the vector genome.[45,46] In mice, ZVex induced robust polyfunctional effector and memory CD8$^+$ T cell responses with prophylactic and therapeutic effects in infectious disease and tumor challenge models after single injection.[45,65] ZVex is currently being evaluated in phase 1 and 2 cancer therapy studies in humans.

Taken together, our data support further development of a conserved mosaic HIV-1 immunogen-based strategy, utilizing heterologous prime-boost approaches with the DC-targeted, integration-deficient lentiviral vector platform ZVex and MVA and/or ChAdOx1 vectors. For the tHIVconsvX as well as other immunogens, we envisage a panel of vectors delivering the same transgene, which will allow a personalized delivery avoiding known pre-existing anti-vector immunity, multiple heterologous boosts for low responders, a maintenance of protective levels of immunity over a prolonged period of time, and more flexibility for combining future effective T and B cell vaccines into one regimen for the best HIV-1 control. The multiple safety features of the ZVex vaccine delivery technology and its T cell immunogenicity make the ZVex platform a very suitable member of such a vector panel for use in both the HIV-1-negative healthy population as part of prevention strategies and in HIV-1-positive patients for HIV-1 cure. For the best preventive immunization, vaccines inducing effective T cells will have to be combined with those eliciting broadly neutralizing antibodies.[66]

MATERIALS AND METHODS

Synthetic Genes for tHIVconsv1 and tHIVconsv2

DNA fragments carrying the tHIVconsv1 and tHIVconsv2 open reading frames were synthesized (Life Technologies) using humanized codons and were preceded by a consensus Kozak sequence at −6 nucleotides to maximize protein expression.[34]

Construction of ZVex.tHIVconsv1 and ZVex.tHIVconsv2 Lentiviral Vector Vaccines

Transfer vector genomes encoding tHIVconsv1 and tHIVconsv2 were constructed by PCR amplification of DNA fragments carrying the tHIVconsv synthetic genes using forward and reverse primers that containing Age1 and EcoR1 restriction sites, respectively. ID-LV was produced as described previously using the ZVex (also known as VP02) platform.[45,46,65] Briefly, ZVex ID-LV was produced via transient transfection of 293T cells with five plasmids: (1) the transfer vector that encodes the ZVex genome and tHIVconsv1 or tHIVconsv2, (2) a modified rev-independent gagpol transcript, (3) accessory protein Rev from HIV-1, (4) accessory protein Vpx from SIVmac, and (5) the SinVar1 (E1001) envelop glycoprotein modified variant derived from Sindbis virus. Harvested vector supernatants were filtered (0.22 μm) and concentrated by centrifugation, followed by benzonase digestion, centrifugation through a sucrose cushion, and resuspension in a formulation Tris-HCl buffer containing 50 mM L-arginine, 5% sucrose. Vector preparations were aliquoted and stored at −80°C until use.

Mice and Immunization Regimens

Six-week-old female BALB/c mice were purchased from Harlan Laboratories and housed at the University of Oxford Functional Genomics Facility. Groups of animals were immunized intramuscularly as indicated in Table S1. In the combined regimens of mosaics 1 and 2, half a dose for each vaccine type was used injecting mosaic 1 and mosaic 2 into the left and right hind quadriceps, respectively.

Figure 4. Cross-Recognition of Epitope Variants following Prime-Boost Vaccinations

Groups of BALB/c mice received vaccines vectored by ZVex (Z), MVA (M), and ChAdOx1 (C) (note that each vaccine modality delivered both mosaic 1 and mosaic 2 together) or empty ZVex without any transgene (Ze) in prime-boost regimens (Table S1) and were euthanized 1 week later. Frequencies of splenocytes recognizing tHIVconsvX peptide pools P1 or P4 indicated above were determined in IFN-γ ELISPOT (A and C) and intracellular cytokine staining (B) assays. The Kruskal-Wallis test (ANOVA) was used for each peptide pool to determine the approximate p values shown above the graph, followed by multiple comparisons of vaccine regimens with Z-Z corrected by the Dunn test. The asterisk indicates $p < 0.05$. (C) Mice were immunized using regimens involving only C or M alone and ZVex without any HIV-1-derived insert and their splenocytes were tested against all tHIVconsvX-derived peptide pools P1-P10 in an IFN-γ ELISPOT assay. (D) Splenocytes from mice immunized as in (A) were also tested for recognition of peptide pools P1 and P4, two 15-mer peptide variants as included in each of the pools, two optimal-length epitope peptides as present in the two mosaic immunogens, and their two additional known variants present in the LANL-HSD. Results are presented as the median and interquartile range, whereby n = 5 in (A), (B), and (D) and n = 4 in (C).

Mice were euthanized between 1 and 2 weeks after the last vaccination. All procedures and care were approved by the University of Oxford local research ethics committee and conformed strictly to the UK Home Office Guidelines under the Animals (Scientific Procedures) Act 1986. Experiments were conducted under Project License 30/3387 held by T.H.

Peptides

Over 90% pure 15-mer peptides overlapping by 11 amino acids (15/11) spanning the entire six conserved regions of the tHIVconsv1 and tHIVconsv2 immunogens were used. A total of 401 peptides were assembled into 10 pools P1–P10 of between 34 and 47 peptides each in a way that variant peptides were always present in the same pool. Individual peptides were dissolved in DMSO at a concentration of 20 mg/ml and stored at −80°C. Working stocks of 4 mg/ml were prepared by diluting 20 mg/ml stocks with PBS. Peptides were used in assays at a final concentration of 1.5 μg/ml.

IFN-γ ELISPOT Assay

The ELISPOT assay was performed using the Mouse IFN-γ ELISPOT kit (Mabtech) according to the manufacturer's instructions. Immune splenocytes were collected and tested separately from individual mice. Spots were visualized using sequential applications of a biotin-conjugated secondary anti-IFN-γ monoclonal antibody (mAb) (R4-6A2, Rat IgG1), an alkaline phosphatase, and a chromogenic substrate (Bio-Rad) and were counted using the AID ELISPOT Reader System (Autoimmun Diagnostika).

Intracellular Cytokine Staining

Cytokine production by splenocytes from immunized mice was assessed by intracellular cytokine staining as described previously.[34] Briefly, splenocytes were stimulated with tHIVconsvX-derived 15/11 peptides assembled into 10 pools, P1–P10, for 90 min at 37°C and then for an additional 5 hr in the presence of brefeldin A (Golgiplug; BD Biosciences) to prevent cytokine secretion. Cells were surface stained with anti-CD8-R-phycoerythrin (PE)-Cy5 red (eBioscience) antibodies and LIVE/DEAD fixable aqua dead cell stain (Invitrogen) and then permeabilized and incubated with various combinations of anti-IL-2-fluorescein isothiocyanate (FITC), anti-CD107a-PE, anti-TNF-α-antigen-presenting cell (APC), and anti-IFN-γ-V450 monoclonal antibodies (BioLegend). Samples were acquired on an LSR II flow cytometer (BD Biosciences) and data were analyzed using FlowJo software (version 9.5.2; Tree Star). Plurifunctionality was assessed using Simplified Presentation of Incredibly Complex Evaluations (SPICE) software (National Institute of Allergy and Infectious Diseases [NIAID]).

Statistical Analysis

Statistical analyses were performed using Graph Pad Prism software (version 6). Responses were assumed to be non-Gaussian in distribution; thus, results are presented as medians (ranges). Multiple comparisons were performed using the Kruskal-Wallis test with the Dunn multiple comparison post-test for non-parametric data. A p value < 0.05 was considered significant.

SUPPLEMENTAL INFORMATION

Supplemental Information includes one figure and one table and can be found with this article online at http://dx.doi.org/10.1016/j.ymthe.2016.12.004.

AUTHOR CONTRIBUTIONS

T.H., J.H.t.M, and A.J.M. conceived the experiments; E.G.W., B.O., P.B., J.A., D.B., J.H.t.M., and T.H. designed and/or carried out the experiments and analyzed the data; and T.H. wrote the article. All authors edited the manuscript.

CONFLICTS OF INTEREST

T.H. and A.J.M. are the inventors on Patent Cooperation Treaty (PCT) application no. PCT/US2014/058422 concerning the tHIVconsvX immunogen. J.H.t.M., J.A., and P.B. are full-time employees and shareholders of Immune Design. D.B. is a member of the scientific advisory board and a shareholder of Immune Design.

ACKNOWLEDGMENTS

The work is jointly funded by the UK Medical Research Council and the UK Department for International Development (DFID) under the MRC/DFID concordat agreement (MRC G1001757 and MR/N023668/1) and by Immune Design Corp. This project has received funding, in part, from the European Union Horizon 2020 Research and Innovation Programme EAVI2020 under a grant agreement (681137). T.H. and A.J.M. are Jenner Institute investigators.

REFERENCES

1. Rappuoli, R. (2011). The challenge of developing universal vaccines. F1000 Med. Rep. *3*, 16.
2. Walker, B., and McMichael, A. (2012). The T-cell response to HIV. Cold Spring Harb. Perspect. Med. *2*, 1–19.
3. Appay, V., Douek, D.C., and Price, D.A. (2008). CD8+ T cell efficacy in vaccination and disease. Nat. Med. *14*, 623–628.
4. Bihl, F., Frahm, N., Di Giammarino, L., Sidney, J., John, M., Yusim, K., Woodberry, T., Sango, K., Hewitt, H.S., Henry, L., et al. (2006). Impact of HLA-B alleles, epitope binding affinity, functional avidity, and viral coinfection on the immunodominance of virus-specific CTL responses. J. Immunol. *176*, 4094–4101.
5. Frahm, N., Korber, B.T., Adams, C.M., Szinger, J.J., Draenert, R., Addo, M.M., Feeney, M.E., Yusim, K., Sango, K., Brown, N.V., et al. (2004). Consistent cytotoxic-T-lymphocyte targeting of immunodominant regions in human immunodeficiency virus across multiple ethnicities. J. Virol. *78*, 2187–2200.
6. Maness, N.J., Valentine, L.E., May, G.E., Reed, J., Piaskowski, S.M., Soma, T., Furlott, J., Rakasz, E.G., Friedrich, T.C., Price, D.A., et al. (2007). AIDS virus specific CD8+ T lymphocytes against an immunodominant cryptic epitope select for viral escape. J. Exp. Med. *204*, 2505–2512.
7. Ferrari, G., Korber, B., Goonetilleke, N., Liu, M.K., Turnbull, E.L., Salazar-Gonzalez, J.F., Hawkins, N., Self, S., Watson, S., Betts, M.R., et al. (2011). Relationship between functional profile of HIV-1 specific CD8 T cells and epitope variability with the selection of escape mutants in acute HIV-1 infection. PLoS Pathog. *7*, e1001273.
8. Goonetilleke, N., Liu, M.K., Salazar-Gonzalez, J.F., Ferrari, G., Giorgi, E., Ganusov, V.V., Keele, B.F., Learn, G.H., Turnbull, E.L., Salazar, M.G., et al.; CHAVI Clinical Core B (2009). The first T cell response to transmitted/founder virus contributes to the control of acute viremia in HIV-1 infection. J. Exp. Med. *206*, 1253–1272.
9. Li, F., Finnefrock, A.C., Dubey, S.A., Korber, B.T., Szinger, J., Cole, S., McElrath, M.J., Shiver, J.W., Casimiro, D.R., Corey, L., and Self, S.G. (2011). Mapping HIV-1 vaccine induced T-cell responses: bias towards less-conserved regions and potential impact on vaccine efficacy in the Step study. PLoS One *6*, e20479.

10. Liu, M.K., Hawkins, N., Ritchie, A.J., Ganusov, V.V., Whale, V., Brackenridge, S., Li, H., Pavlicek, J.W., Cai, F., Rose-Abrahams, M., et al.; CHAVI Core B (2013). Vertical T cell immunodominance and epitope entropy determine HIV-1 escape. J. Clin. Invest. *123*, 380–393.

11. Pala, P., Serwanga, J., Watera, C., Ritchie, A.J., Moodie, Z., Wang, M., Goonetilleke, N., Birabwa, E., Hughes, P., Senkaali, D., et al.; Center for HIV/AIDS Vaccine Immunology (2013). Quantitative and qualitative differences in the T cell response to HIV in uninfected Ugandans exposed or unexposed to HIV-infected partners. J. Virol. *87*, 9053–9063.

12. Ternette, N., Block, P.D., Sánchez-Bernabéu, Á., Borthwick, N., Pappalardo, E., Abdul-Jawad, S., Ondondo, B., Charles, P.D., Dorrell, L., Kessler, B.M., and Hanke, T. (2015). Early kinetics of the HLA class I-associated peptidome of MVA.HIVconsv-infected cells. J. Virol. *89*, 5760–5771.

13. Ternette, N., Yang, H., Partridge, T., Llano, A., Cedeño, S., Fischer, R., Charles, P.D., Dudek, N.L., Mothe, B., Crespo, M., et al. (2016). Defining the HLA class I-associated viral antigen repertoire from HIV-1-infected human cells. Eur. J. Immunol. *46*, 60–69.

14. Borrow, P., Lewicki, H., Hahn, B.H., Shaw, G.M., and Oldstone, M.B. (1994). Virus-specific CD8+ cytotoxic T-lymphocyte activity associated with control of viremia in primary human immunodeficiency virus type 1 infection. J. Virol. *68*, 6103–6110.

15. Boutwell, C.L., Rolland, M.M., Herbeck, J.T., Mullins, J.I., and Allen, T.M. (2010). Viral evolution and escape during acute HIV-1 infection. J. Infect. Dis. *202* (*Suppl 2*), S309–S314.

16. Brander, C., Goulder, P.J., Luzuriaga, K., Yang, O.O., Hartman, K.E., Jones, N.G., Walker, B.D., and Kalams, S.A. (1999). Persistent HIV-1-specific CTL clonal expansion despite high viral burden post in utero HIV-1 infection. J. Immunol. *162*, 4796–4800.

17. Goulder, P.J., Brander, C., Annamalai, K., Mngqundaniso, N., Govender, U., Tang, Y., He, S., Hartman, K.E., O'Callaghan, C.A., Ogg, G.S., et al. (2000). Differential narrow focusing of immunodominant human immunodeficiency virus gag-specific cytotoxic T-lymphocyte responses in infected African and caucasoid adults and children. J. Virol. *74*, 5679–5690.

18. Koup, R.A., Safrit, J.T., Cao, Y., Andrews, C.A., McLeod, G., Borkowsky, W., Farthing, C., and Ho, D.D. (1994). Temporal association of cellular immune responses with the initial control of viremia in primary human immunodeficiency virus type 1 syndrome. J. Virol. *68*, 4650–4655.

19. Streeck, H., Jolin, J.S., Qi, Y., Yassine-Diab, B., Johnson, R.C., Kwon, D.S., Addo, M.M., Brumme, C., Routy, J.P., Little, S., et al. (2009). Human immunodeficiency virus type 1-specific CD8+ T-cell responses during primary infection are major determinants of the viral set point and loss of CD4+ T cells. J. Virol. *83*, 7641–7648.

20. Carlson, J.M., Du, V.Y., Pfeifer, N., Bansal, A., Tan, V.Y., Power, K., Brumme, C.J., Kreimer, A., DeZiel, C.E., Fusi, N., et al. (2016). Impact of pre-adapted HIV transmission. Nat. Med. *22*, 606–613.

21. Hanke, T. (2014). Conserved immunogens in prime-boost strategies for the next-generation HIV-1 vaccines. Expert Opin. Biol. Ther. *14*, 601–616.

22. Haynes, B.F., Shaw, G.M., Korber, B., Kelsoe, G., Sodroski, J., Hahn, B.H., Borrow, P., and McMichael, A.J. (2016). HIV-host interactions: implications for vaccine design. Cell Host Microbe *19*, 292–303.

23. Létourneau, S., Im, E.-J., Mashishi, T., Brereton, C., Bridgeman, A., Yang, H., Dorrell, L., Dong, T., Korber, B., McMichael, A.J., and Hanke, T. (2007). Design and pre-clinical evaluation of a universal HIV-1 vaccine. PLoS One *2*, e984.

24. Rolland, M., Nickle, D.C., and Mullins, J.I. (2007). HIV-1 group M conserved elements vaccine. PLoS Pathog. *3*, e157.

25. Carlson, J.M., Schaefer, M., Monaco, D.C., Batorsky, R., Claiborne, D.T., Prince, J., Deymier, M.J., Ende, Z.S., Klatt, N.R., DeZiel, C.E., et al. (2014). HIV transmission. Selection bias at the heterosexual HIV-1 transmission bottleneck. Science *345*, 1254031.

26. Claiborne, D.T., Prince, J.L., Scully, E., Macharia, G., Micci, L., Lawson, B., Kopycinski, J., Deymier, M.J., Vanderford, T.H., Nganou-Makamdop, K., et al. (2015). Replicative fitness of transmitted HIV-1 drives acute immune activation, proviral load in memory CD4+ T cells, and disease progression. Proc. Natl. Acad. Sci. USA *112*, E1480–E1489.

27. Deng, K., Pertea, M., Rongvaux, A., Wang, L., Durand, C.M., Ghiaur, G., Lai, J., McHugh, H.L., Hao, H., Zhang, H., et al. (2015). Broad CTL response is required to clear latent HIV-1 due to dominance of escape mutations. Nature *517*, 381–385.

28. Ferguson, A.L., Mann, J.K., Omarjee, S., Ndung'u, T., Walker, B.D., and Chakraborty, A.K. (2013). Translating HIV sequences into quantitative fitness landscapes predicts viral vulnerabilities for rational immunogen design. Immunity *38*, 606–617.

29. Kunwar, P., Hawkins, N., Dinges, W.L., Liu, Y., Gabriel, E.E., Swan, D.A., Stevens, C.E., Maenza, J., Collier, A.C., Mullins, J.I., et al. (2013). Superior control of HIV-1 replication by CD8+ T cells targeting conserved epitopes: implications for HIV vaccine design. PLoS One *8*, e64405.

30. Ahmed, T., Borthwick, N.J., Gilmour, J., Hayes, P., Dorrell, L., and Hanke, T. (2016). Control of HIV-1 replication in vitro by vaccine-induced human CD8(+) T cells through conserved subdominant Pol epitopes. Vaccine *34*, 1215–1224.

31. Borthwick, N., Ahmed, T., Ondondo, B., Hayes, P., Rose, A., Ebrahimsa, U., Hayton, E.J., Black, A., Bridgeman, A., Rosario, M., et al. (2014). Vaccine-elicited human T cells recognizing conserved protein regions inhibit HIV-1. Mol. Ther. *22*, 464–475.

32. Hayton, E.J., Rose, A., Ibrahimsa, U., Del Sorbo, M., Capone, S., Crook, A., Black, A.P., Dorrell, L., and Hanke, T. (2014). Safety and tolerability of conserved region vaccines vectored by plasmid DNA, simian adenovirus and modified vaccinia virus Ankara administered to human immunodeficiency virus type 1-uninfected adults in a randomized, single-blind phase I trial. PLoS One *9*, e101591.

33. Mutua, G., Farah, B., Langat, R., Indangasi, J., Ogola, S., Onsembe, B., Kopycinski, J.T., Hayes, P., Borthwick, N.J., Ashraf, A., et al. (2016). Broad HIV-1 inhibition in vitro by vaccine-elicited CD8(+) T cells in African adults. Mol. Ther. Methods Clin. Dev. *3*, 16061.

34. Ondondo, B., Murakoshi, H., Clutton, G., Abdul-Jawad, S., Wee, E.G., Gatanaga, H., Oka, S., McMichael, A.J., Takiguchi, M., Korber, B., and Hanke, T. (2016). Novel conserved-region T-cell mosaic vaccine with high global HIV-1 coverage is recognized by protective responses in untreated infection. Mol. Ther. *24*, 832–842.

35. Abdul-Jawad, S., Ondondo, B., van Hateren, A., Gardner, A., Elliott, T., Korber, B., and Hanke, T. (2016). Increased valency of conserved-mosaic vaccines enhances the breadth and depth of epitope recognition. Mol. Ther. *24*, 375–384.

36. Barouch, D.H., O'Brien, K.L., Simmons, N.L., King, S.L., Abbink, P., Maxfield, L.F., Sun, Y.H., La Porte, A., Riggs, A.M., Lynch, D.M., et al. (2010). Mosaic HIV-1 vaccines expand the breadth and depth of cellular immune responses in rhesus monkeys. Nat. Med. *16*, 319–323.

37. Hulot, S.L., Korber, B., Giorgi, E.E., Vandergrift, N., Saunders, K.O., Balachandran, H., Mach, L.V., Lifton, M.A., Pantaleo, G., Tartaglia, J., et al. (2015). Comparison of immunogenicity in rhesus macaques of transmitted-founder, HIV-1 group M consensus, and trivalent mosaic envelope vaccines formulated as a DNA prime, NYVAC, and envelope protein boost. J. Virol. *89*, 6462–6480.

38. Santra, S., Liao, H.X., Zhang, R., Muldoon, M., Watson, S., Fischer, W., Theiler, J., Szinger, J., Balachandran, H., Buzby, A., et al. (2010). Mosaic vaccines elicit CD8+ T lymphocyte responses that confer enhanced immune coverage of diverse HIV strains in monkeys. Nat. Med. *16*, 324–328.

39. Mothe, B., Llano, A., Ibarrondo, J., Daniels, M., Miranda, C., Zamarreño, J., Bach, V., Zuniga, R., Pérez-Álvarez, S., Berger, C.T., et al. (2011). Definition of the viral targets of protective HIV-1-specific T cell responses. J. Transl. Med. *9*, 208.

40. Murakoshi, H., Akahoshi, T., Koyanagi, M., Chikata, T., Naruto, T., Maruyama, R., Tamura, Y., Ishizuka, N., Gatanaga, H., Oka, S., and Takiguchi, M. (2015). Clinical control of HIV-1 by cytotoxic T cells specific for multiple conserved epitopes. J. Virol. *89*, 5330–5339.

41. Butler, N.S., Nolz, J.C., and Harty, J.T. (2011). Immunologic considerations for generating memory CD8 T cells through vaccination. Cell. Microbiol. *13*, 925–933.

42. Dull, T., Zufferey, R., Kelly, M., Mandel, R.J., Nguyen, M., Trono, D., and Naldini, L. (1998). A third-generation lentivirus vector with a conditional packaging system. J. Virol. *72*, 8463–8471.

43. He, Y., and Falo, L.D., Jr. (2007). Lentivirus as a potent and mechanistically distinct vector for genetic immunization. Curr. Opin. Mol. Ther. *9*, 439–446.

44. Zufferey, R., Dull, T., Mandel, R.J., Bukovsky, A., Quiroz, D., Naldini, L., and Trono, D. (1998). Self-inactivating lentivirus vector for safe and efficient in vivo gene delivery. J. Virol. *72*, 9873–9880.

45. Odegard, J.M., Kelley-Clarke, B., Tareen, S.U., Campbell, D.J., Flynn, P.A., Nicolai, C.J., Slough, M.M., Vin, C.D., McGowan, P.J., Nelson, L.T., et al. (2015). Virological and preclinical characterization of a dendritic cell targeting, integration-deficient lentiviral vector for cancer immunotherapy. J. Immunother. *38*, 41–53.

46. Tareen, S.U., Kelley-Clarke, B., Nicolai, C.J., Cassiano, L.A., Nelson, L.T., Slough, M.M., Vin, C.D., Odegard, J.M., Sloan, D.D., Van Hoeven, N., et al. (2014). Design of a novel integration-deficient lentivector technology that incorporates genetic and posttranslational elements to target human dendritic cells. Mol. Ther. *22*, 575–587.

47. Fischer, W., Perkins, S., Theiler, J., Bhattacharya, T., Yusim, K., Funkhouser, R., Kuiken, C., Haynes, B., Letvin, N.L., Walker, B.D., et al. (2007). Polyvalent vaccines for optimal coverage of potential T-cell epitopes in global HIV-1 variants. Nat. Med. *13*, 100–106.

48. Barouch, D.H., Pau, M.G., Custers, J.H., Koudstaal, W., Kostense, S., Havenga, M.J., Truitt, D.M., Sumida, S.M., Kishko, M.G., Arthur, J.C., et al. (2004). Immunogenicity of recombinant adenovirus serotype 35 vaccine in the presence of pre-existing anti-Ad5 immunity. J. Immunol. *172*, 6290–6297.

49. Bridgeman, A., Roshorm, Y., Lockett, L.J., Xu, Z.-Z., Hopkins, R., Shaw, J., Both, G.W., and Hanke, T. (2009). Ovine atadenovirus, a novel and highly immunogenic vector in prime-boost studies of a candidate HIV-1 vaccine. Vaccine *28*, 474–483.

50. McCoy, K., Tatsis, N., Korioth-Schmitz, B., Lasaro, M.O., Hensley, S.E., Lin, S.W., Li, Y., Giles-Davis, W., Cun, A., Zhou, D., et al. (2007). Effect of preexisting immunity to adenovirus human serotype 5 antigens on the immune responses of nonhuman primates to vaccine regimens based on human- or chimpanzee-derived adenovirus vectors. J. Virol. *81*, 6594–6604.

51. Buchbinder, S.P., Mehrotra, D.V., Duerr, A., Fitzgerald, D.W., Mogg, R., Li, D., Gilbert, P.B., Lama, J.R., Marmor, M., Del Rio, C., et al.; Step Study Protocol Team (2008). Efficacy assessment of a cell-mediated immunity HIV-1 vaccine (the Step Study): a double-blind, randomised, placebo-controlled, test-of-concept trial. Lancet *372*, 1881–1893.

52. Flynn, N.M., Forthal, D.N., Harro, C.D., Judson, F.N., Mayer, K.H., and Para, M.F.; rgp120 HIV Vaccine Study Group (2005). Placebo-controlled phase 3 trial of a recombinant glycoprotein 120 vaccine to prevent HIV-1 infection. J. Infect. Dis. *191*, 654–665.

53. Pitisuttithum, P., Gilbert, P., Gurwith, M., Heyward, W., Martin, M., van Griensven, F., Hu, D., Tappero, J.W., and Choopanya, K.; Bangkok Vaccine Evaluation Group (2006). Randomized, double-blind, placebo-controlled efficacy trial of a bivalent recombinant glycoprotein 120 HIV-1 vaccine among injection drug users in Bangkok, Thailand. J. Infect. Dis. *194*, 1661–1671.

54. Akpogheneta, O. (2011). Correlates of protection and HIV vaccine development. Lancet Infect. Dis. *11*, 814–815.

55. Robert-Guroff, M. (2007). Replicating and non-replicating viral vectors for vaccine development. Curr. Opin. Biotechnol. *18*, 546–556.

56. Hill, A.V., Reyes-Sandoval, A., O'Hara, G., Ewer, K., Lawrie, A., Goodman, A., Nicosia, A., Folgori, A., Colloca, S., Cortese, R., et al. (2010). Prime-boost vectored malaria vaccines: progress and prospects. Hum. Vaccin. *6*, 78–83.

57. McShane, H., and Hill, A. (2005). Prime-boost immunisation strategies for tuberculosis. Microbes Infect. *7*, 962–967.

58. Paris, R.M., Kim, J.H., Robb, M.L., and Michael, N.L. (2010). Prime-boost immunization with poxvirus or adenovirus vectors as a strategy to develop a protective vaccine for HIV-1. Expert Rev. Vaccines *9*, 1055–1069.

59. Beignon, A.S., Mollier, K., Liard, C., Coutant, F., Munier, S., Rivière, J., Souque, P., and Charneau, P. (2009). Lentiviral vector-based prime/boost vaccination against AIDS: pilot study shows protection against Simian immunodeficiency virus SIVmac251 challenge in macaques. J. Virol. *83*, 10963–10974.

60. Asefa, B., Korokhov, N., and Lemiale, F. (2010). Heterologous HIV-based lentiviral/adenoviral vectors immunizations result in enhanced HIV-specific immunity. Vaccine *28*, 3617–3624.

61. Banasik, M.B., and McCray, P.B., Jr. (2010). Integrase-defective lentiviral vectors: progress and applications. Gene Ther. *17*, 150–157.

62. Goyvaerts, C., and Breckpot, K. (2015). Pros and cons of antigen-presenting cell targeted tumor vaccines. J. Immunol. Res. *2015*, 785634.

63. Yang, L., Yang, H., Rideout, K., Cho, T., Joo, K.I., Ziegler, L., Elliot, A., Walls, A., Yu, D., Baltimore, D., and Wang, P. (2008). Engineered lentivector targeting of dendritic cells for in vivo immunization. Nat. Biotechnol. *26*, 326–334.

64. Berger, G., Goujon, C., Darlix, J.L., and Cimarelli, A. (2009). SIVMAC Vpx improves the transduction of dendritic cells with nonintegrative HIV-1-derived vectors. Gene Ther. *16*, 159–163.

65. Albershardt, T.C., Campbell, D.J., Parsons, A.J., Slough, M.M., ter Meulen, J., and Berglund, P. (2016). Lv305, a dendritic cell-targeting integration-deficient Zvex™-based lentiviral vector encoding Ny-Eso-1, induces potent anti-tumor immune response. Mol Ther Oncolytics, Published online March 30, 2016. http://dx.doi.org/10.1038/mto.2016.10.

66. McMichael, A.J., and Haynes, B.F. (2012). Lessons learned from HIV-1 vaccine trials: new priorities and directions. Nat. Immunol. *13*, 423–427.

YMTHE, Volume *25*

Supplemental Information

HIV-1 Conserved Mosaics Delivered by Regimens with Integration-Deficient DC-Targeting Lentiviral Vector Induce Robust T Cells

Edmund G. Wee, Beatrice Ondondo, Peter Berglund, Jacob Archer, Andrew J. McMichael, David Baltimore, Jan H. ter Meulen, and Tomáš Hanke

Supplementary Table S1. Immunization regimens

	Grp	Regimen	n	Prime (Week 0)		Boost (Week 3)		Boost (Week 6)	
**Figure 2**	1	Z	4	ZVex.tHIVconsv1	$5x10^{8}$ gc	-	-	-	-
BALB/c	2	Z	4	ZVex.tHIVconsv1	$5x10^{9}$ gc	-	-	-	-
	3	Z	4	ZVex.tHIVconsv1	$1x10^{10}$ gc	-	-	-	-
	4	Z	4	ZVex.tHIVconsv2	$5x10^{8}$ gc	-	-	-	-
	5	Z	4	ZVex.tHIVconsv2	$5x10^{9}$ gc	-	-	-	-
	6	Z	4	ZVex.tHIVconsv2	$1x10^{10}$ gc	-	-	-	-
**Figure 3**	1	ZZ	5	ZVex.tHIVconsv1	$2.5x10^{9}$ gc	ZVex.tHIVconsv1	$2.5x10^{9}$ gc	-	-
BALB/c				ZVex.tHIVconsv2	$2.5x10^{9}$ gc	ZVex.tHIVconsv2	$2.5x10^{9}$ gc		
	2	ZM	5	ZVex.tHIVconsv1	$2.5x10^{9}$ gc	MVA.tHIVconsv3	$5x10^{5}$ PFU	-	-
				ZVex.tHIVconsv2	$2.5x10^{9}$ gc	MVA.tHIVconsv4	$5x10^{5}$ PFU		
	3	ZC	5	ZVex.tHIVconsv1	$2.5x10^{9}$ gc	ChAdOx1.tHIVconsv5	$1.4x10^{6}$ IU	-	-
				ZVex.tHIVconsv2	$2.5x10^{9}$ gc	ChAdOx1.tHIVconsv6	$1.4x10^{6}$ IU		
**Figure 4**	1	ZZ	5	ZVex.tHIVconsv1	$2.5x10^{9}$ gc	ZVex.tHIVconsv1	$2.5x10^{9}$ gc	-	-
**a, b & d**				ZVex.tHIVconsv2	$2.5x10^{9}$ gc	ZVex.tHIVconsv2	$2.5x10^{9}$ gc		
BALB/c	2	ZM	5	ZVex.tHIVconsv1	$2.5x10^{9}$ gc	MVA.tHIVconsv3	$5x10^{5}$ PFU	-	-
				ZVex.tHIVconsv2	$2.5x10^{9}$ gc	MVA.tHIVconsv4	$5x10^{5}$ PFU		
	3	ZC	5	ZVex.tHIVconsv1	$2.5x10^{9}$ gc	ChAdOx1.tHIVconsv5	$1.4x10^{6}$ IU	-	-
				ZVex.tHIVconsv2	$2.5x10^{9}$ gc	ChAdOx1.tHIVconsv6	$1.4x10^{6}$ IU		
	4	CZ	5	ChAdOx1.tHIVconsv5	$1.4x10^{6}$ IU	ZVex.tHIVconsv1	$2.5x10^{9}$ gc	-	-
				ChAdOx1.tHIVconsv6	$1.4x10^{6}$ IU	ZVex.tHIVconsv2	$2.5x10^{9}$ gc		
	5	MZ	5	MVA.tHIVconsv3	$5x10^{5}$ PFU	ZVex.tHIVconsv1	$2.5x10^{9}$ gc	-	-
				MVA.tHIVconsv4	$5x10^{5}$ PFU	ZVex.tHIVconsv2	$2.5x10^{9}$ gc		
	6	CM	5	ChAdOx1.tHIVconsv5	$1.4x10^{6}$ IU	MVA.tHIVconsv3	$5x10^{5}$ PFU	-	-
				ChAdOx1.tHIVconsv6	$1.4x10^{6}$ IU	MVA.tHIVconsv4	$5x10^{5}$ PFU		
**Figure 4c**	1	ZZ	4	ZVex.tHIVconsv1	$2.5x10^{9}$ gc	ZVex.tHIVconsv1	$2.5x10^{9}$ gc	-	-
				ZVex.tHIVconsv2	$2.5x10^{9}$ gc	ZVex.tHIVconsv2	$2.5x10^{9}$ gc		
BALB/c	2	ZM	4	ZVex.tHIVconsv1	$2.5x10^{9}$ gc	MVA.tHIVconsv3	$5x10^{5}$ PFU	-	-
				ZVex.tHIVconsv2	$2.5x10^{9}$ gc	MVA.tHIVconsv4	$5x10^{5}$ PFU		
	3	ZC	4	ZVex.tHIVconsv1	$2.5x10^{9}$ gc	ChAdOx1.tHIVconsv5	$1.4x10^{6}$ IU	-	-
				ZVex.tHIVconsv2	$2.5x10^{9}$ gc	ChAdOx1.tHIVconsv6	$1.4x10^{6}$ IU		
	4	-M	4	-	-	MVA.tHIVconsv3	$5x10^{5}$ PFU	-	-
						MVA.tHIVconsv4	$5x10^{5}$ PFU		
	5	-C	4	-	-	ChAdOx1.tHIVconsv5	$1.4x10^{6}$ IU	-	-
						ChAdOx1.tHIVconsv6	$1.4x10^{6}$ IU		
	6	ZeZe	4	ZVex empty	$2.5x10^{9}$ gc	ZVex empty	$2.5x10^{9}$ gc	-	-
				ZVex empty	$2.5x10^{9}$ gc	ZVex empty	$2.5x10^{9}$ gc		
**Figure 5**	1	ZM	7	ZVex.tHIVconsv1	$2.5x10^{9}$ gc	MVA.tHIVconsv3	$5x10^{5}$ PFU	-	-
CD1-Swiss				ZVex.tHIVconsv2	$2.5x10^{9}$ gc	MVA.tHIVconsv4	$5x10^{5}$ PFU		
	2	CM	7	ChAdOx1.tHIVconsv5	$1.4x10^{6}$ IU	MVA.tHIVconsv3	$5x10^{5}$ PFU	-	-
				ChAdOx1.tHIVconsv6	$1.4x10^{6}$ IU	MVA.tHIVconsv4	$5x10^{5}$ PFU		
	3	ZCM	7	ZVex.tHIVconsv1	$2.5x10^{9}$ gc	ChAdOx1.tHIVconsv5	$1.4x10^{6}$ IU	MVA.tHIVconsv3	$5x10^{5}$ PFU
				ZVex.tHIVconsv2	$2.5x10^{9}$ gc	ChAdOx1.tHIVconsv6	$1.4x10^{6}$ IU	MVA.tHIVconsv4	$5x10^{5}$ PFU

gc – genome copies; IU – infection unit; PFU – plaque-forming unit

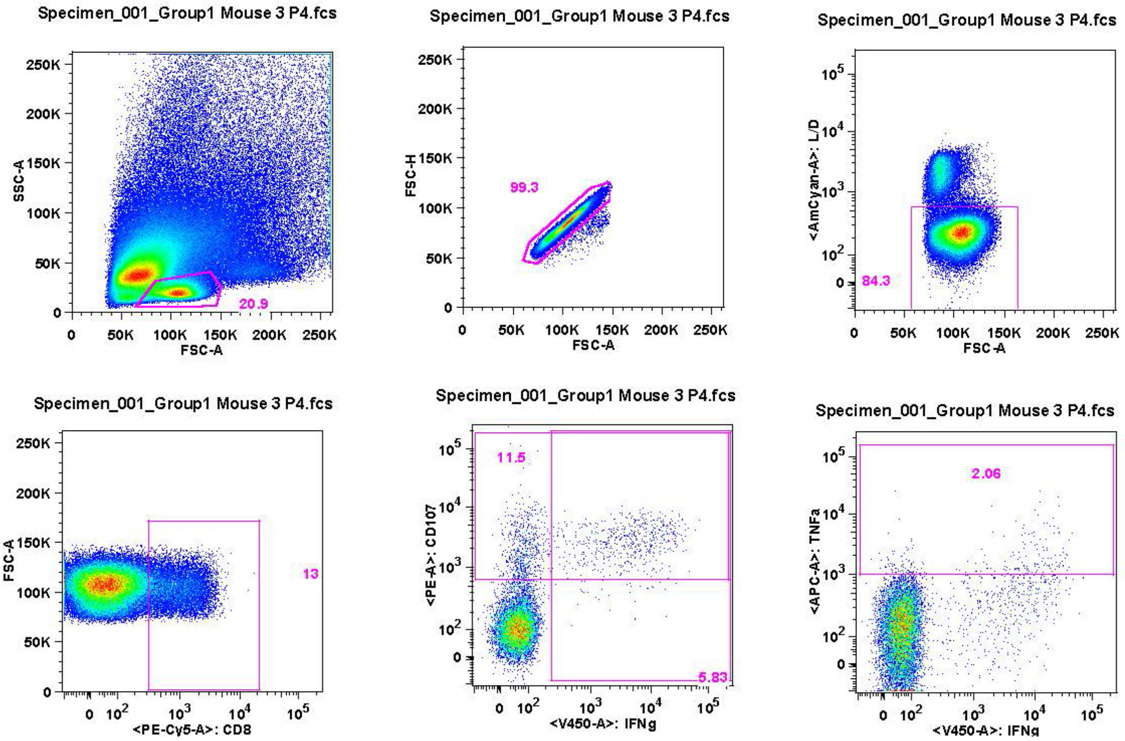

Supplementary Figure S1 Gating strategy for **Figure 3** Representative FACS plots show the gating strategy for assessing co-expression of IFN-γ, TNF-α, IL-2 and CD107a.
